# When and how to treat isolated visceral artery dissections – protocol proposal

**DOI:** 10.1590/1677-5449.202300662

**Published:** 2023-06-30

**Authors:** Paulo Eduardo Ocke Reis, Arno Von Ristow

**Affiliations:** 1 Universidade Federal Fluminense – UFF, Rio de Janeiro, RJ, Brasil.; 2 Clínica Vascular Ocke Reis, Rio de Janeiro, RJ, Brasil.; 3 Academia Nacional de Medicina, Rio de Janeiro, RJ, Brasil.; 4 Pontifícia Universidade Católica do Rio de Janeiro – PUC-Rio, Rio de Janeiro, RJ, Brasil.

Arterial dissections refer to the abnormal, and usually abrupt, formation of a tear involving the inside wall of an artery. As the tear becomes larger, it forms a small pouch, which is called a “false lumen.” Accumulation of blood inside this false lumen can generate thrombi or otherwise block the blood flow, leading either to local complications – of which rupture is the most threatening – or to local thrombosis or downstream embolization, generating a variety of symptoms.^1^

Although visceral artery dissections can occur in any splanchnic artery, only small series have been published, reporting single center experience of spontaneous isolated dissection of the celiac artery (SIDCA) or of the superior mesenteric artery (SIDSMA). These represent the major types of spontaneous visceral artery dissection. Furthermore, no quantitative meta-analyses of SIDCA or SIDSMA are available, as stated by Wang in 2018.^1^ Fortunately, these are rare events and, in most cases, are asymptomatic. For these reasons, there is no consensus in the current literature on how to approach these entities. The diagnostic method most used currently is computerized tomography, because it can show the false lumen and the true lumen separately, clearly showing the intimal flap.^2,3^

**Figure 1:**
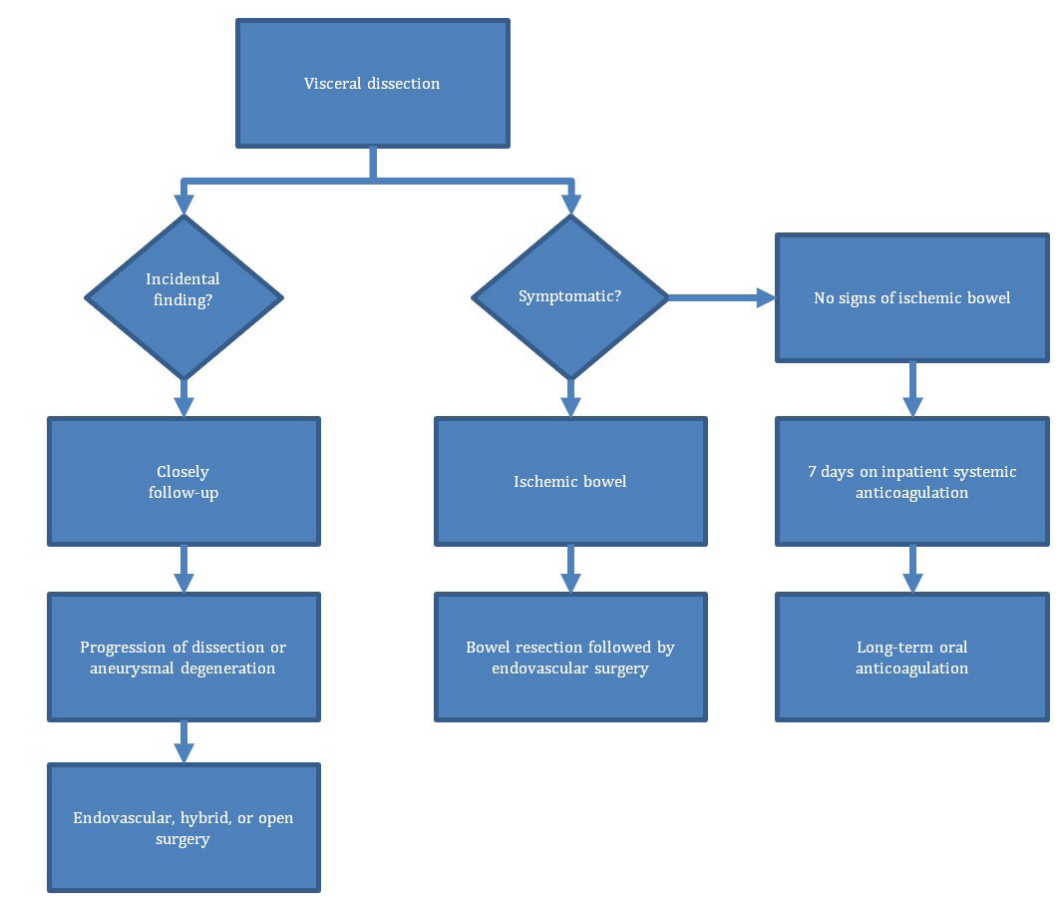
Isolated Visceral Artery Dissection Protocol. Based on data from Sakamoto et al.^4^, Ristow et al.^5^ and Cardoso et al.^6^

Protocols based on clinical and imaging studies to support the decision about the best time to intervene in patients with SIDCA and SIDSMA have been proposed for certain segments, but do not encompass the whole spectrum of this entity. Here, we present a modified algorithm based on publications by Sakamoto et al. in 2007, Ristow et al. in 2010, and Cardoso et al. in 2013 (Figure 1).^4-6^

Algorithm description, from left to right: If SIDCA and/or SIDSMA are diagnosed as incidental findings in asymptomatic individuals, observation without intervention is indicated, with close follow-up with imaging studies for the first year. If aneurysmal progression or degeneration occurs, treatment can be with endovascular, hybrid, or open surgery, depending on the case. In the center of the algorithm, in symptomatic patients with suspicion of intra-abdominal hemorrhage or bowel ischemia, open surgery with bowel resection as indicated; Endovascular or hybrid surgery should be promptly performed through a clean field if indicated. On the right side of our protocol, in symptomatic patients with no signs of visceral ischemia, hospital admission with at least 7 days of systemic anticoagulation is mandatory. Watchful attention to symptoms and/or signs of organ ischemia is mandatory. Long term oral anticoagulation is indicated because of the risk of progression and potential clinical worsening.

How to treat SIDCA and SIDSMA? Conservative, open, hybrid, or endovascular approaches may each be the best option, depending on the evaluation and clinical evolution of each case. We must keep in mind that the goal of interventional treatment is complete remodeling of the dissection, when feasible.^1-8^ In symptomatic patients, surgical treatment, preferably endovascular, should be considered.^9,10^ So, in conclusion, in our experience, follow-up with an adequate imaging study should be the rule for all patients. Conservative and pharmacological treatment is sufficient in most cases. In cases of failure of conservative treatment, endovascular or hybrid treatment is feasible in most situations. Open surgery, possibly preceded by laparoscopy, should be reserved for cases in which irreversible bowel compromise is suspected.
